# Investigating longitudinal associations of hair cortisol and cortisone with cognitive functioning and dementia

**DOI:** 10.1038/s41598-022-25143-z

**Published:** 2022-11-30

**Authors:** Cornelia Santoso, David Stuckler, Andreas Ihle

**Affiliations:** 1 Faculty of Public Health, University of Debrecen, Debrecen, Hungary; 2 Dondena Centre for Research On Social Dynamics, Bocconi University, Milan, Italy; 3 Department of Psychology, University of Geneva, 1205 Geneva, Switzerland; 4 Center for the Interdisciplinary Study of Gerontology and Vulnerability, University of Geneva, 1205 Geneva, Switzerland; 5 Swiss National Centre of Competence in Research LIVES—Overcoming Vulnerability: Life Course Perspectives, 1015 Lausanne, Switzerland

**Keywords:** Biomarkers, Risk factors, Psychiatric disorders, Medical research, Biomarkers, Epidemiology

## Abstract

We rigorously investigated potential longitudinal associations of hair cortisol and cortisone with verbal memory, time orientation, and dementia, adjusting for sociodemographic and health confounders. Data from the English Longitudinal Study of Ageing wave 6–9 (6-year follow-up, covering 4399 persons aged 50+) were analysed using linear random effects and cox regression models. In unadjusted models, hair cortisol was associated with worsened verbal memory (β 0.19; SE 0.08), but not with time orientation (β 0.02; SE 0.01), or dementia (β 0.07; SE 0.16). Hair cortisone was associated with worsened verbal memory (β 0.74; SE 0.14) and time orientation (β 0.06; SE 0.02), but not with dementia (β 0.47; SE 0.28). However, in the fully adjusted models, neither hair cortisol nor cortisone was associated with verbal memory, time orientation, or dementia. Consistent with prior studies, we found that more advanced age was associated with worsened verbal memory (β 0.15; SE 0.01), time orientation (β 0.01; SE 0.00), and dementia risk (β 0.11; SE 0.02). Our rigorous analyses did not detect robust associations of neither hair cortisol nor cortisone with cognitive functioning or dementia across 6 years. More detailed insights into potential mechanisms are discussed.

## Introduction

An important, however to date not fully answered question concerns whether chronic exposure to psychological and biological stressors does negatively affect cognitive functioning^1,2^. There is a considerable body of research suggesting that high levels of stress hormones may lead to high metabolic demand, as well as having effects on the brain. The latter is because glucocorticoid (GC) stress hormones bind with GC receptors that are located in several parts of the brain. For example, the GC receptors in the hippocampus are suggested to play a role in episodic short-term and long term memory, and prospective memory^2–5^, while those in the prefrontal cortex may affect working memory and executive functions^2,3^.

One major stress hormone is cortisol and cortisone. Yet, it has proven difficult to measure. Past studies have sought to measure cortisol by capturing GC levels in blood, urine, or saliva at a single time point. However, these methods may not effectively capture chronic exposure to cortisol, as these values vary considerably, depending on circadian rhythm, pulsatile secretion, and situational factors (e.g. acute stressors)^3,6^. Studies using these methods have also produced mixed results, especially those of longitudinal studies with cognitively healthy adults at baseline^7^. For example, a study among male twins in the United States found that increased levels of area-under-the-curve salivary cortisol was associated with lower visual–spatial memory, executive function, and processing speed^8^. Meanwhile, a study in France found that elevated salivary cortisol level was associated with visual memory decline in women but not in men^7,9^. Similarly, a study among postmenopausal women in California found that an increased blood cortisol level was associated with reduced category fluency but not with other cognitive aspects^7,10^. Another study among older adults in Rotterdam found that serum total cortisol and free cortisol levels were not associated with cognitive decline, measured by Mini-Mental State Examination (MMSE)^8,11^.

More recently, cortisol levels have been measured from hair samples. This method is more valid for expressing long-term cortisol exposure over several months^2,3,6,12^. They have also been found not to be affected by diurnal rhythmicity, acute stress, and hair-related factors, including hair treatments^3,6,13^. Furthermore, it is relevant to additionally measure hair cortisone levels. While hair cortisol has frequently been used to assess chronic stress, its inactive form, cortisone, is understudied^2,14^. Hair cortisone could serve as an additional promising biomarker for evaluating the amount of GC. Cortisone can be transformed into cortisol by the 11β-hydroxysteroid dehydrogenase (11β-HSD) type 1 enzyme. Meanwhile, the 11β-HSD type 2 enzyme can reverse the process^2,15^. Evaluating cortisone as a potential risk factor for cognitive impairment may be worthwhile. The 11β-HSD1 expression in the hippocampus and forebrain has been suggested to rise in the aging brain, serving as a potential mechanism underlying the brain’s vulnerability to excessive GC levels^2,16^.

To date, there are only very few studies investigating the association of hair cortisol and cortisone and cognitive performance in non-clinical adults. Those were only based on cross-sectional studies and produced mixed results. For example, a study among older adults in Ireland found that increased levels of hair cortisol and cortisone were associated with poorer verbal memory and global cognition^2^. On the other hand, a study among older adults in Spain found that a low level of hair cortisol was associated with worse working memory and verbal memory^17^. Another study among middle-aged working adults in Germany found no association between hair cortisol levels and any of the cognitive domains^3^. Yet, to the best of our knowledge, there is no study so far investigating these associations on a longitudinal time frame among non-clinical adults.

To address this important gap, we analysed data from the English Longitudinal Study of Ageing (ELSA), which measured hair cortisol and cortisone of older persons in England in 2012 and had the latest follow-up in 2018. The aim of our study was to rigorously examine the longitudinal associations of hair cortisol and cortisone with cognitive performance and dementia among older adults in England, adjusting for potential sociodemographic and health confounders.

## Results

### Characteristics of the samples

Table 1 summarises the baseline characteristics of our analytical sample based on the distribution of hair cortisol at wave 6. For the purpose of descriptive statistics, hair cortisol was categorised into low (< 31.1 pg/ml) and high (≥ 31.1 pg/ml)^18,19^ [note that hair cortisol was analysed as a continuous variable in the main models]. Briefly, the mean (± SD) age of the sample was 67.7 (± 9.0) years. Around 67% was female, had white ethnicity, had intermediate education level, were retired, were married or with a partner, were former smoker, drinking alcohol daily or almost daily, had moderate physical activity level, and did not have any heart diseases, stroke, diabetes, hypertension, nor high cholesterol.

**Table 1:** Baseline characteristics of the sample based on the distribution of hair cortisol.

	Total	Low cortisol^a^	High cortisol^a^
		N (%)	N (%)
**Outcomes**			
Worsened verbal memory at wave 6^c^	8.9 (± 3.4)	8.9 (± 3.4)	9.0 (± 3.2)
Worsened time orientation at wave 6^c^	0.2 (± 0.4)	0.2 (± 0.4)	0.2 (± 0.4)
**Cumulative dementia cases**			
No	3453 (97.0)	2965 (85.9)	488 (14.1)
Yes	107 (3.0)	90 (84.1)	17 (15.9)
**Baseline covariates at wave 6**^**b**^			
Age^c^	67.7 (± 9.0)	67.5 (± 9.0)	69.0 (± 8.8)
**Gender**			
Female	2935 (66.7)	2534 (86.3)	401 (13.7)
Male	1467 (33.3)	1255 (85.5)	212 (14.5)
**Ethnic**			
Non-white	82 (1.9)	74 (90.2)	8 (9.8)
White	4320 (98.1)	3715 (86.0)	605 (14.0)
**Education**			
No qualification	1015 (23.1)	859 (84.6)	156 (15.4)
Intermediate	1980 (45.0)	1717 (86.7)	263 (13.3)
Higher education	1407 (32.0)	1213 (86.2)	194 (13.8)
**Wealth**			
Quintile 1	676 (17.5)	587 (86.8)	89 (13.2)
Quintile 2	757 (19.6)	644 (85.1)	113 (14.9)
Quintile 3	801 (20.7)	694 (86.6)	107 (13.4)
Quintile 4	814 (21.0)	692 (85.0)	122 (15.0)
Quintile 5	824 (21.3)	706 (85.7)	118 (14.3)
**Employment**			
Retired	2650 (68.4)	2246 (84.8)	404 (15.2)
Employed	1186 (30.6)	1049 (88.4)	137 (11.6)
Unemployed	36 (0.9)	28 (77.8)	8 (22.2)
**Marital status**			
Married/partner	2551 (65.9)	2186 (85.7)	365 (14.3)
Separated/divorce	465 (12.0)	409 (88.0)	56 (12.0)
Widowed	644 (16.6)	543 (84.3)	101 (15.7)
Single	212 (5.5)	185 (87.3)	27 (12.7)
**Smoking**			
Never	1571 (40.6)	1360 (86.6)	211 (13.4)
Former	1930 (49.8)	1634 (84.7)	296 (15.3)
Current	371 (9.6)	329 (88.7)	42 (11.3)
**Alcohol consumption**			
Never	486 (12.6)	428 (88.1)	58 (11.9)
< 2x/month	1156 (29.9)	963 (83.3)	193 (16.7)
1–2/week	887 (22.9)	764 (86.1)	123 (13.9)
Daily, almost daily	1343 (34.7)	1168 (87)	175 (13)
**Physical activity**			
None/light	752 (19.4)	629 (83.6)	123 (16.4)
Moderate	1905 (49.2)	1640 (86.1)	265 (13.9)
High	1215 (31.4)	1054 (86.7)	161 (13.3)
Depression score^c^	1.2 (± 1.7)	1.2 (± 1.7)	1.3 (± 1.8)
**Heart diseases**			
No	3152 (81.4)	2715 (86.1)	437 (13.9)
Yes	720 (18.6)	608 (84.4)	112 (15.6)
**Stroke**			
No	3732 (96.4)	3212 (86.1)	520 (13.9)
Yes	140 (3.6)	111 (79.3)	29 (20.7)
**Diabetes**			
No	3513 (90.7)	3026 (86.1)	487 (13.9)
Yes	359 (9.3)	297 (82.7)	62 (17.3)
**Hypertension**			
No	2246 (58.0)	1959 (87.2)	287 (12.8)
Yes	1626 (42.0)	1364 (83.9)	262 (16.1)
**High cholesterol**			
No	2399 (62.0)	2097 (87.4)	302 (12.6)
Yes	1473 (38.0)	1226 (83.2)	247 (16.8)
**Assay phase**			
Phase 1	2453 (55.7)	2027 (82.6)	426 (17.4)
Phase 2	1949 (44.3)	1762 (90.4)	187 (9.6)
**Hair treatment**			
No	2638 (59.9)	2264 (85.8)	374 (14.2)
Yes	1764 (40.1)	1525 (86.5)	239 (13.5)

^a^For the purpose of descriptive statistics, hair cortisol was categorised into low (< 31.1 pg/ml) and high (≥ 31.1 pg/ml)^18,19^.

^b^Baseline covariates at wave 6 were derived from the sample used to investigate the association between hair cortisol and worsened verbal memory.

^c^Continuous variables were expressed as mean (± SD).

A similar summary for hair cortisone can be seen in Supplementary Table S2, with hair cortisone categorised based on the median value into low (< 6.9 pg/ml) and high (≥ 6.9 pg/ml) [note that hair cortisone was analysed as a continuous variable in the main models].

### Associations of hair cortisol and cortisone with cognitive functioning and dementia

First, we investigated the associations of hair cortisol and cortisone with cognitive functioning and dementia in unadjusted regression models. Table 2 shows the unadjusted results of all our independent variables with cognitive performance and dementia. Hair cortisol was associated with worsened verbal episodic memory (β 0.19; P-value 0.020), but not with worsened time orientation (β 0.02; P-value 0.054), or dementia (β 0.07; P-value 0.687). Hair cortisone was associated with worsened verbal episodic memory (β 0.74; P-value < 0.001) and worsened time orientation (β 0.06; P-value < 0.001), but not with dementia (β 0.47; P-value 0.091).

**Table 2:** Unadjusted regression models of hair cortisol and cortisone as the exposures and cognitive performance and dementia as the outcomes.

	Worsened verbal memory^a^	Worsened time orientation^a^	Dementia^b^
	ß (SE)		
Log hair cortisol	0.19 (0.08)*	0.02 (0.01)	0.07 (0.16)
Log hair cortisone	0.74 (0.14)***	0.06 (0.02)***	0.47 (0.28)
**Covariates**^**c**^			
Age^d^	0.17 (0.00)***	0.01 (0.00)***	0.13 (0.01)***
**Gender**			
Female	Ref	Ref	Ref
Male	0.75 (0.10)***	0.04 (0.01)**	0.12 (0.20)
**Ethnic**			
Non-white	Ref	Ref	Ref
White	− 1.09 (0.36)**	− 0.07 (0.04)	− 1.00 (0.46)*
**Education**			
No qualification	Ref	Ref	Ref
Intermediate	− 2.01 (0.12)***	− 0.08 (0.02)***	− 0.78 (0.23)***
Higher education	− 2.70 (0.13)***	− 0.10 (0.02)***	− 0.85 (0.25)***
**Wealth**			
Quintile 1	Ref	Ref	Ref
Quintile 2	− 0.52 (0.11)***	− 0.02 (0.02)	0.12 (0.27)
Quintile 3	− 0.79 (0.11)***	− 0.04 (0.02)*	− 0.12 (0.29)
Quintile 4	− 1.24 (0.11)***	− 0.05 (0.02)**	− 0.78 (0.35)*
Quintile 5	− 1.64 (0.12)***	− 0.09 (0.02)***	− 0.41 (0.31)
**Employment**			
Retired	Ref	Ref	Ref
Employed	− 1.18 (0.08)***	− 0.10 (0.01)***	− 2.31 (0.46)***
Unemployed	− 0.77 (0.33)*	− 0.06 (0.06)	− 0.39 (1.00)
**Marital status**			
Married/partner	Ref	Ref	Ref
Separated/divorce	0.08 (0.13)	− 0.02 (0.02)	− 0.37 (0.38)
Widowed	1.34 (0.11)***	0.09 (0.01)***	0.94 (0.21)***
Single	− 0.14 (0.20)	0.01 (0.03)	− 0.94 (0.72)
**Smoking**			
Never	Ref	Ref	Ref
Former	0.41 (0.10)***	0.02 (0.01)	0.27 (0.20)
Current	0.42 (0.15)**	0.04 (0.02)	− 0.37 (0.44)
**Alcohol consumption**			
Never	Ref	Ref	Ref
< 2x/month	− 1.13 (0.10)***	− 0.08 (0.02)***	− 0.66 (0.28)*
1–2/week	− 1.49 (0.11)***	− 0.10 (0.02)***	− 0.60 (0.29)*
Daily, almost daily	− 1.60 (0.12)***	− 0.11 (0.02)***	− 0.93 (0.28)**
**Physical activity**			
None/light	Ref	Ref	Ref
Moderate	− 0.97 (0.07)***	− 0.06 (0.01)***	− 0.89 (0.22)***
High	− 1.45 (0.08)***	− 0.12 (0.01)***	− 1.35 (0.28)***
Depression^d^	0.17 (0.02)***	0.01 (0.00)***	0.15 (0.05)**
**Heart diseases**			
No	Ref	Ref	Ref
Yes	0.76 (0.09)***	0.06 (0.01)***	0.77 (0.21)***
**Stroke**			
No	Ref	Ref	Ref
Yes	2.13 (0.18)***	0.15 (0.03)***	1.65 (0.28)***
**Diabetes**			
No	Ref	Ref	Ref
Yes	0.92 (0.13)***	0.05 (0.02)**	0.60 (0.27)*
**Hypertension**			
No	Ref	Ref	Ref
Yes	0.79 (0.08)***	0.02 (0.01)*	0.37 (0.19)
**High cholesterol**			
No	Ref	Ref	Ref
Yes	0.59 (0.08)***	0.01 (0.01)	0.54 (0.19)**
**Assay phase**			
Phase 1	Ref	Ref	Ref
Phase 2	0.11 (0.10)	0.02 (0.01)	0.48 (0.19)*
**Hair treatment**			
No	Ref	Ref	Ref
Yes	− 0.69 (0.10)***	− 0.04 (0.01)**	− 0.32 (0.21)

*P < 0.05, **P < 0.01, ***P < 0.001.

^a^Linear random effect models were used to investigate the associations of hair cortisol and cortisone with worsened verbal memory and time orientation.

^b^Cox regression model was used to investigate the associations of hair cortisol and cortisone with dementia.

^c^The models were derived from the sample used to investigate the associations of hair cortisol with worsened verbal memory, worsened time orientation, and dementia.

^d^Age and depression score were treated as continuous variables.

Next, we turned to adjusted regression models. Table 3 shows the adjusted results of hair cortisol and other covariates with cognitive performance and dementia. In the linear random effect model, hair cortisol was not associated with worsened verbal episodic memory (β − 0.01; P-value 0.929) or worsened time orientation (β 0.01; P-value 0.262). In the Cox regression model, cortisol was not associated with dementia (β − 0.04; P-value 0.811).

**Table 3:** Adjusted regression models of hair cortisol as the exposure and cognitive performance and dementia as the outcomes.

	Worsened verbal memory^a^	Worsened time orientation^a^	Dementia^b^
	ß (SE)		
Log hair cortisol	− 0.01 (0.07)	0.01 (0.01)	− 0.04 (0.17)
**Covariates**			
Age^c^	0.15 (0.01)***	0.01 (0.00)***	0.11 (0.02)***
**Gender**			
Female	Ref	Ref	Ref
Male	1.24 (0.11)***	0.07 (0.02)***	0.13 (0.26)
**Ethnic**			
Non-white	Ref	Ref	Ref
White	− 1.26 (0.29)***	− 0.08 (0.04)	− 1.34 (0.49)**
**Education**			
No qualification	Ref	Ref	Ref
Intermediate	− 0.99 (0.11)***	− 0.02 (0.02)	− 0.07 (0.24)
Higher education	− 1.65 (0.12)***	− 0.03 (0.02)	− 0.08 (0.28)
**Wealth**			
Quintile 1	Ref	Ref	Ref
Quintile 2	− 0.17 (0.10)	− 0.00 (0.02)	0.12 (0.28)
Quintile 3	− 0.27 (0.10)*	− 0.01 (0.02)	0.04 (0.31)
Quintile 4	− 0.50 (0.11)***	− 0.01 (0.02)	− 0.54 (0.38)
Quintile 5	− 0.74 (0.12)***	− 0.04 (0.02)	0.00 (0.35)
**Employment**			
Retired	Ref	Ref	Ref
Employed	0.09 (0.08)	− 0.02 (0.01)	− 0.78 (0.50)
Unemployed	0.21 (0.31)	0.01 (0.06)	1.34 (1.05)
**Marital status**			
Married/partner	Ref	Ref	Ref
Separated/divorce	− 0.09 (0.11)	− 0.02 (0.02)	− 0.26 (0.39)
Widowed	− 0.12 (0.10)	− 0.00 (0.02)	− 0.21 (0.25)
Single	− 0.09 (0.17)	0.01 (0.03)	− 1.14 (0.73)
**Smoking**			
Never	Ref	Ref	Ref
Former	0.01 (0.08)	0.01 (0.01)	0.12 (0.21)
Current	0.30 (0.13)*	0.04 (0.02)*	− 0.00 (0.46)
**Alcohol consumption**			
Never	Ref	Ref	Ref
< 2x/month	− 0.54 (0.10)***	− 0.04 (0.02)*	− 0.03 (0.30)
1–2/week	− 0.62 (0.11)***	− 0.04 (0.02)*	0.21 (0.31)
Daily, almost daily	− 0.64 (0.11)***	− 0.05 (0.02)*	0.00 (0.33)
**Physical activity**			
None/light	Ref	Ref	Ref
Moderate	− 0.42 (0.07)***	− 0.02 (0.01)	− 0.27 (0.23)
High	− 0.61 (0.08)***	− 0.05 (0.01)***	− 0.38 (0.31)
Depression^c^	0.10 (0.02)***	0.01 (0.00)*	0.08 (0.05)
**Heart diseases**			
No	Ref	Ref	Ref
Yes	− 0.04 (0.08)	0.01 (0.01)	0.21 (0.22)
**Stroke**			
No	Ref	Ref	Ref
Yes	0.93 (0.16)***	0.08 (0.03)**	0.79 (0.29)**
**Diabetes**			
No	Ref	Ref	Ref
Yes	0.11 (0.11)	0.01 (0.02)	0.19 (0.29)
**Hypertension**			
No	Ref	Ref	Ref
Yes	− 0.10 (0.08)	− 0.03 (0.01)*	− 0.36 (0.21)
**High cholesterol**			
No	Ref	Ref	Ref
Yes	− 0.01 (0.07)	− 0.02 (0.01)	0.24 (0.20)
**Assay phase**			
Phase 1	Ref	Ref	Ref
Phase 2	0.41 (0.08)***	0.04 (0.01)**	0.36 (0.21)
**Hair treatment**			
No	Ref	Ref	Ref
Yes	0.11 (0.10)	0.01 (0.01)	− 0.08 (0.25)

*P < 0.05, **P < 0.01, ***P < 0.001.

^a^Linear random effect models were used to investigate the associations between hair cortisol and worsened verbal memory and time orientation.

^b^Cox regression model was used to investigate the association between hair cortisol and dementia.

^c^Age and depression score were treated as continuous variables.

Table 4 shows the adjusted results of hair cortisone and other covariates with cognitive performance and dementia. In the linear random effect model, hair cortisone was not associated with worsened verbal episodic memory (β 0.21; P-value 0.074) or worsened time orientation (β 0.03; P-value 0.071). In the Cox regression model, hair cortisone was not associated with dementia (β 0.28; P-value 0.375). Furthermore, consistent with prior studies, we found that advanced age was associated with worsened verbal episodic memory and time orientation and dementia. Smoking and depression were associated with worsened verbal episodic memory and time orientation (Tables 3, 4).

**Table 4:** Adjusted regression models of hair cortisone as the exposure and cognitive performance and dementia as the outcomes.

	Worsened verbal memory^a^	Worsened time orientation^a^	Dementia^b^
	ß (SE)		
Log hair cortisone	0.21 (0.12)	0.03 (0.02)	0.28 (0.31)
**Covariates**			
Age^c^	0.15 (0.01)***	0.01 (0.00)***	0.11 (0.02)***
**Gender**			
Female	Ref	Ref	Ref
Male	1.21 (0.10)***	0.06 (0.02)***	0.03 (0.25)
**Ethnic**			
Non-white	Ref	Ref	Ref
White	− 1.14 (0.28)***	− 0.07 (0.04)	− 1.26 (0.49)*
**Education**			
No qualification	Ref	Ref	Ref
Intermediate	− 0.98 (0.10)***	− 0.03 (0.02)	0.01 (0.23)
Higher education	− 1.66 (0.11)***	− 0.04 (0.02)*	0.01 (0.27)
**Wealth**			
Quintile 1	Ref	Ref	Ref
Quintile 2	− 0.20 (0.09)*	− 0.01 (0.02)	0.04 (0.27)
Quintile 3	− 0.30 (0.10)**	− 0.02 (0.02)	0.00 (0.30)
Quintile 4	− 0.50 (0.11)***	− 0.02 (0.02)	− 0.44 (0.36)
Quintile 5	− 0.72 (0.11)***	− 0.04 (0.02)*	− 0.06 (0.34)
**Employment**			
Retired	Ref	Ref	Ref
Employed	0.12 (0.08)	− 0.02 (0.01)	− 0.85 (0.50)
Unemployed	0.22 (0.30)	− 0.01 (0.05)	1.16 (1.04)
**Marital status**			
Married/partner	Ref	Ref	Ref
Separated/divorce	− 0.08 (0.11)	− 0.02 (0.02)	− 0.25 (0.37)
Widowed	− 0.12 (0.10)	− 0.01 (0.02)	− 0.24 (0.24)
Single	− 0.04 (0.17)	0.00 (0.03)	− 1.16 (0.73)
**Smoking**			
Never	Ref	Ref	Ref
Former	0.05 (0.08)	0.01 (0.01)	0.16 (0.21)
Current	0.34 (0.13)**	0.04 (0.02)*	− 0.07 (0.46)
**Alcohol consumption**			
Never	Ref	Ref	Ref
< 2x/month	− 0.54 (0.09)***	− 0.04 (0.02)**	− 0.12 (0.28)
1–2/week	− 0.64 (0.10)***	− 0.04 (0.02)*	0.14 (0.30)
Daily, almost daily	− 0.67 (0.11)***	− 0.05 (0.02)**	− 0.17 (0.31)
**Physical activity**			
None/light	Ref	Ref	Ref
Moderate	− 0.39 (0.06)***	− 0.02 (0.01)	− 0.26 (0.22)
High	− 0.56 (0.08)***	− 0.05 (0.01)***	− 0.43 (0.30)
Depression^c^	0.10 (0.02)***	0.01 (0.00)*	0.09 (0.05)
**Heart diseases**			
No	Ref	Ref	Ref
Yes	− 0.04 (0.08)	0.00 (0.01)	0.14 (0.21)
**Stroke**			
No	Ref	Ref	Ref
Yes	0.86 (0.16)***	0.07 (0.03)*	0.75 (0.28)**
**Diabetes**			
No	Ref	Ref	Ref
Yes	0.11 (0.11)	0.02 (0.02)	0.32 (0.26)
**Hypertension**			
No	Ref	Ref	Ref
Yes	− 0.06 (0.07)	− 0.03 (0.01)*	− 0.31 (0.20)
**High cholesterol**			
No	Ref	Ref	Ref
Yes	− 0.02 (0.07)	− 0.02 (0.01)	0.26 (0.19)
**Assay phase**			
Phase 1	Ref	Ref	Ref
Phase 2	0.34 (0.08)***	0.03 (0.01)**	0.30 (0.20)
**Hair treatment**			
No	Ref	Ref	Ref
Yes	0.11 (0.10)	0.01 (0.01)	− 0.13 (0.24)

*P < 0.05, **P < 0.01, ***P < 0.001.

^a^Linear random effect models were used to investigate the associations between hair cortisone and worsened verbal memory and time orientation.

^b^Cox regression model was used to investigate the association between hair cortisone and dementia.

^c^Age and depression score were treated as continuous variables.

**Figure 1:**
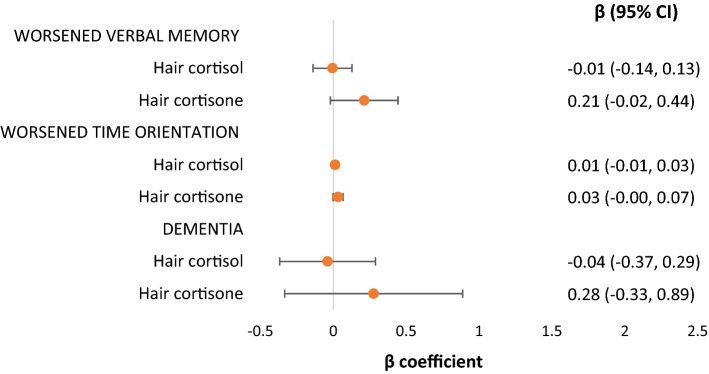
Associations of hair cortisol and cortisone and cognitive function and dementia in fully adjusted models.

Figure 1 summarises the associations of hair cortisol and cortisone with cognitive performance and dementia in the fully adjusted models. Neither hair cortisol nor cortisone had significant associations with worsened verbal episodic memory and time orientation and dementia.

## Discussion

Our study rigorously investigated potential longitudinal associations of hair cortisol and cortisone with cognitive functioning and dementia among older adults in England. Hair cortisol and cortisone levels were not found to be longitudinally associated with verbal episodic memory, time orientation, or dementia.

Our novel longitudinal findings that hair cortisol was not associated with cognitive functioning or dementia were in line with cross-sectional studies in Germany^1,3^, but not with other cross-sectional studies in Ireland^2^, South Africa^20^, or Spain^17^. The variation in these findings may be due to the differences in the study design, characteristics of the study sample, such as age, and cognitive domains tested. Those in South Africa^20^ and Spain^17^ are based on cross-sectional studies and small samples, or involved samples of younger adults. Furthermore, another study suggested that the association between cortisol and cognition might only be observed among people with other coexisting vulnerabilities, such as psychiatric illnesses related to chronic stress (e.g. depression) or those with lower education level^1^. Although our study population was relatively similar to the cross-sectional study in Ireland^2^, their study involved a relatively larger proportion of females than our study sample. Furthermore, their findings for the associations of cortisone and immediate-delayed recall were inconsistent across gender and observed only among women. These questions regarding which cognitive domain may be affected by hair cortisone and which study population called for further research^2^.

Our findings are also in contrast with studies investigating acute stress markers using saliva or serum sample and then found inverse relationships between cortisol level and cognitive functioning^7,9,10^. It is suggested that in contrast to such short-term effects of acute stress, long-term cortisol level as a chronic stress marker may have a much less important influence on cognitive performance^3^. A cross-sectional study among older adults in Spain found that while increased hair cortisol was associated with better verbal memory, increased salivary cortisol was associated with worse cognition in the same sample^2,17^. They argued that hair cortisol might interact with diurnal saliva cortisol, by which individuals with lower hair cortisol may be more prone to the negative effect of reduced diurnal change, which is worsened cognitive functioning^3,17^. Furthermore, hair and salivary cortisol may capture different information regarding hypothalamic–pituitary–adrenal (HPA) activities. Hair cortisol serves as an indicator of integrated HPA activity over several months, while salivary cortisol may relate to acute peaks of stress and/or abnormal diurnal patterns, which indicate the regulation of HPA-axis circadian rhythmicity^3,17^.

Alternative explanations of our findings may be that the effect of long-term cortisol on cognition may appear at later ages, requiring a longer follow-up time. For example, a study based on Whitehall II in England found no association between diurnal cortisol and cognitive impairment within a 5-year period^21^ but found an association within a 10-year period^22^.

Finally, while we did not find longitudinal associations of hair cortisol and cortisone with verbal episodic memory and time orientation scores and dementia, further studies might investigate associations with other cognitive domains and with longer follow-up time. It might also be useful to re-analyse clinical trials of pharmacological and non-pharmacological interventions of cortisol-lowering effects to investigate their impact on cognitive functioning and dementia.

Our study has some limitations. First, our analyses were based on unweighted data, and thus they may not be fully representative of the general older population in England. The weighting variables provided by the survey were computed based on the sample that was followed from wave 1, or wave 4, to wave 9^23^, and thus applying this weighting in our analyses (wave 6–9) may cause some deviations from the original population. Second, most behavioural data, such as alcohol consumption, was based on self-reported information, which may be prone to a certain inaccuracy and social desirability bias. However, it might only be the type of non-differential misclassification and will not affect our estimates. Third, we only investigate the cognitive domains of verbal episodic memory and time orientation. It is possible that other cognitive domains may show associations with hair cortisol and cortisone^21^. Fourth, the generalisability of dementia cases in our study should be interpreted with caution as the survey question did not specify the type of dementia. Furthermore, due to a small number of cases, we combined physician-diagnosis of Alzheimer’s disease or dementia, and the IQCODE score (≥ 3.5), to refer to dementia in our study. Fifth, one may argue that any or all confounders in our analyses may contribute to the significant associations between hair cortisol and cortisone and cognition in the unadjusted models. While our study was unable to pinpoint precisely which was the main (or multiple) confounding factor, it did show that age, wealth, and hypertension appeared to attenuate the relationship between hair cortisol and verbal memory to non-significance (see Supplementary Figure S2). Future research would be needed to ascertain the underlying factors which specifically may have driven spurious associations in prior research.

Nevertheless, our study was the very first to rigorously investigate potential longitudinal associations of hair cortisol and cortisone with cognitive ageing in non-clinical adults. Furthermore, the follow-up time of six years in our study was considered adequate and had a large sample size, which increased the precision of our estimates. Adjusting for a large variety of potential sociodemographic and health confounders is a further strength of our study.

## Methods

Following best practices, we adhered to the Strengthening the Reporting of Observational Studies in Epidemiology (STROBE) guidelines for cohort studies^24^.

### Study population

This study used data from the English Longitudinal Study of Ageing (ELSA), a panel study of persons aged 50+ and their partners in England. The survey received ethical approval from the National Health Service (NHS) Research Ethics Committees under the National Research and Ethics Service (NRES). Details of the survey have been described elsewhere^23^. Since this study is a secondary data analysis, a separate ethical approval is not needed.

**Figure 2:**
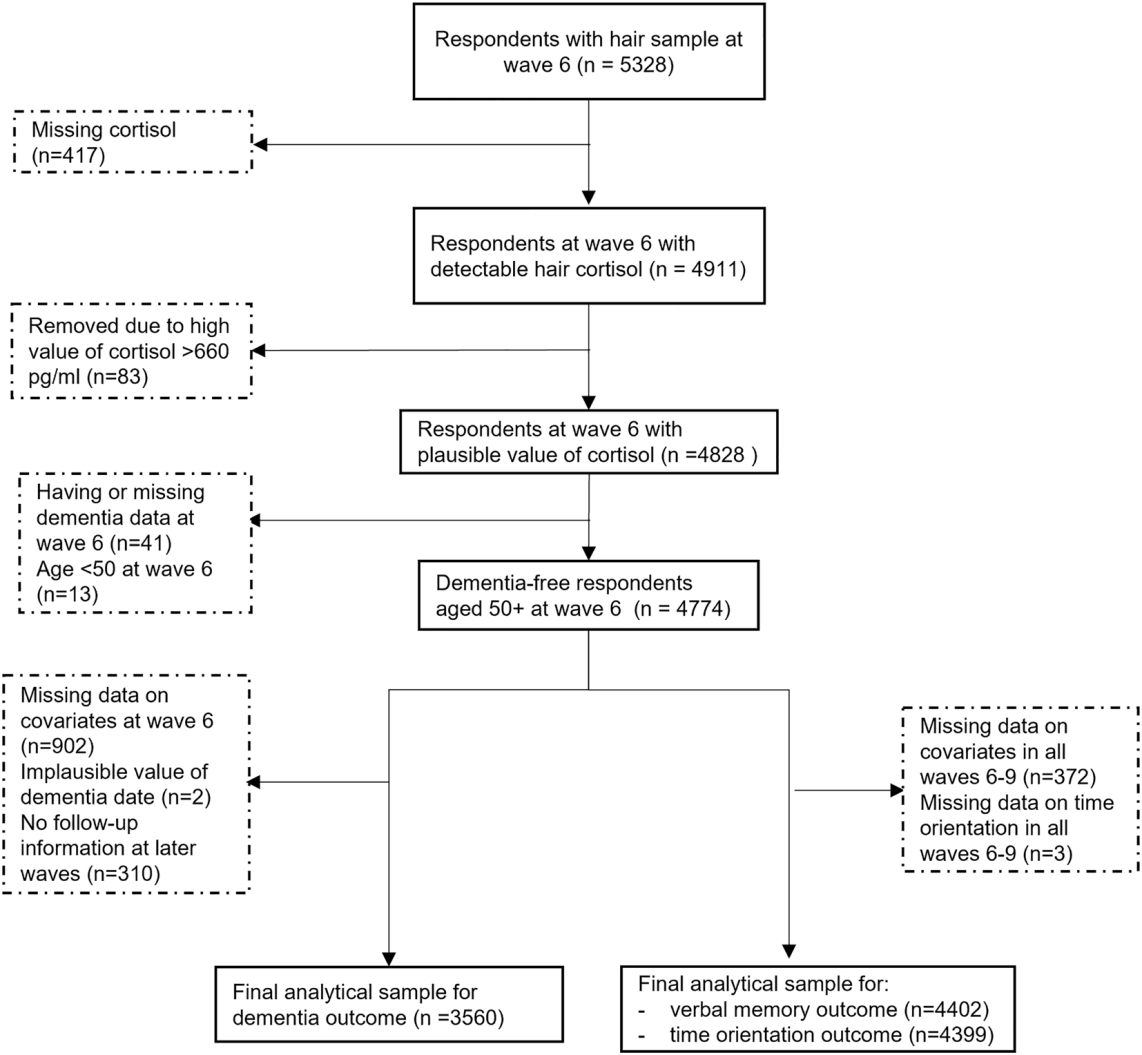
Flowchart of the analytical sample of hair cortisol.

We used data from ELSA wave 6 (2012/3), which was the only wave when the hair sample of the participants was collected^23^, to wave 9 (2018/9), which is the latest wave available at the time of this study. Of a total of 5328 participants with hair samples at wave 6, 4828 had a detectable and plausible value of hair cortisol. After excluding those with dementia at wave 6 and missing data on covariates, the final analytical samples for verbal memory score, time orientation score, and dementia were 4402, 4399, and 3560, respectively. Figure 2 shows the flowchart of the study participants and sample inclusion for hair cortisol. A similar flowchart for hair cortisone can be seen in Supplementary Figure S1.

### Measurements of hair cortisol and cortisone

Hair sampling and hormone assays procedure have been described in detail in the documentation of ELSA wave 6^23^. Briefly, the survey collected hair samples from the participants during the nurse visit, excluding those who were breastfeeding, incapable of sitting with the head remaining still, had a scalp condition that impeded sample collection or had hair length < 2 cm at the posterior vertex. The hair length for steroid extraction was targeted to be 3 cm most proximal to the scalp. Following a wash and steroid extraction, hormone assays was performed using liquid chromatography-mass spectrometry. Considering that the distributions of hair cortisol and cortisone were positively skewed, the log-10 transformation was applied. Hair cortisol with an implausibly high value (> 660 pg/ml) was excluded from the analyses^23^.

### Measurements of cognitive functioning and dementia

Three outcomes were assessed in this study. The first two outcomes related to cognitive functioning that were consistently measured from waves 6 to 9 were verbal episodic memory and time orientation scores. The verbal episodic memory was assessed through immediate and delayed free recall tests. The participants were read a list of ten common unrelated words, with the rate of one word every two seconds. There were four non-overlapping 10-word lists, allowing them to be given distinct lists in different waves^23,25–28^. Details of the word lists can be seen in the Supplementary Table S1. The participants were then instructed to recall them immediately, and following a brief delay (roughly five minutes), during which they were presented with other cognitive tests^26,27^. They were given maximum two minutes to recall them^23,25^. One point was given for each correct word recalled (maximum 10 points for each test). Following previous studies^26–29^, a verbal episodic memory score was calculated as the sum of these immediate and delayed word recall test scores, with the total score ranging from 0 to 20.

The time orientation score was calculated as the sum of four indicators. One point each was given if participants could correctly indicate the following questions: the date, month, year, and day of the week at the time of the interview. The total score ranged from 0 to 4^28,29^. To facilitate easier interpretation regarding worsened cognitive performance across all cognitive functioning and dementia measures, we reverse-coded the scales so that higher scores correspond to worse verbal episodic memory or time orientation scores.

The third outcome was dementia. Following previous studies^30–32^, it was defined by two criteria. The main criterion was based on physician diagnosis for either Alzheimer’s disease or dementia, reported by participants or the informants. The secondary criterion was based on the score of the 16-question Informant Questionnaire on Cognitive Decline in the Elderly (IQCODE), which was completed by the informants. A score of 3.5 or above was used to define dementia. This cut-off point has been shown to have high sensitivity and specificity for detecting dementia cases^30,33^. Overall, there were 107 dementia cases in our study. Of these, 98 (92%) were identified from the main criterion, and 9 (8%) were from the secondary criterion.

### Measurements of covariates

Covariates used in our study included a group of sociodemographic variables, health behaviours, and health and well-being status of the participants. Following previous studies, these covariates were selected a priori as they have been demonstrated to have associations with both cortisol and cognitive function^1,2,10,21,34–44^. Furthermore, as recommended by the survey, hair-related factors, such as hair treatment (i.e. hair dyed or chemically treated) (yes/no) and assay phase (phase 1 or 2) were included as covariates^23^.

The socio-demographic variables included age, gender (male/female), ethnic (white/non-white), marital status (married or in civil partnership/separated or divorce/widowed/never married), education, wealth, and employment status (employed/unemployed/retired). The highest educational attainment of the participants was classified into three categories: low (‘no qualification’), medium (‘NVQ1/CSE other grade equivalent qualification’, ‘NVQ2/GCE O-level equivalent qualification’, ‘NVQ3/GCE A-level equivalent qualification’, ‘foreign/other’), and high (‘higher education below degree’, ‘NVQ4/NVQ5/Degree or equivalent qualification’). Total net wealth, which was the sum of savings, investments, physical wealth and housing wealth after subtracting financial debt and mortgage debt, was used to divide the sample into five wealth quantiles^23^.

Health behaviours included smoking status (never/former/current), frequency of alcohol consumption in the past year (none/less than twice a month/once or twice a week/daily or almost daily), and physical activity. The latter was constructed based on the participants’ answers concerning intensity (light, moderate, vigorous) and frequency (hardly ever or never, one to three times per month, once per week, more than once per week) of physical activity. It was then classified into three categories: none or light activity only/moderate activity at least once weekly/vigorous activity at least once weekly^45^.

Health and well-being status variables included depression, and ever having heart diseases (yes/no), stroke (yes/no), diabetes (yes/no), hypertension (yes/no), or high cholesterol (yes/no). The depression score was calculated as the sum of the 8-item Center for Epidemiologic Studies Depression Scale (CES-D). One point each was given if the participants indicated the following: feeling depressed, feeling everything that they did was an effort, having a restless sleep, feeling lonely, feeling sad, being unhappy, not be able to get going much of the time, and not enjoying life. The total score ranged from 0 to 8, with higher scores indicating worsened symptoms^23^.

### Statistical analysis

We used descriptive statistics to summarise the characteristics of the sample. Considering the differences in the type of outcome variables, we employed two types of statistical models. First, we used linear random effect models to model the associations of hair cortisol and cortisone with the outcomes of verbal episodic memory and time orientation scores from wave 6 to 9. Second, we used cox proportional hazard regression to model the associations of hair cortisol and cortisone at wave 6 with the cumulative dementia cases from wave 7 to 9. Participants with dementia at wave 6 or prior were excluded from the analyses. Time to the development of dementia was calculated in months from the participants’ interview date at wave 6 to the date of dementia diagnosis. In case of unknown date of dementia diagnosis, we used the midpoint of interview dates between waves. Dropped-out participants were censored, with the last interview date as the censor date^31,32^. The Cox regression models included adjustment for baseline covariates at wave 6. All analyses were conducted unweighted in STATA 17.0^46^.

## Supplementary Information

Supplementary Information.

## Data Availability

The English Longitudinal Study of Ageing (ELSA) data can be publicly accessed through UK Data Service portal. Procedure to access the data can be found at: https://www.elsa-project.ac.uk/accessing-elsa-data.
