# Supplementary material for: Investigating longitudinal associations of hair cortisol and cortisone with cognitive functioning and dementia
Source: Sci Rep. 2022 Nov 30;12:20642. doi: 10.1038/s41598-022-25143-z (PMC9712516; doi:10.1038/s41598-022-25143-z)

**Supplementary Information**

**Manuscript title**: Investigating longitudinal associations of hair cortisol and cortisone with cognitive functioning and dementia

**Authors**: Cornelia Santoso, David Stuckler, Andreas Ihle

Supplementary Table S1. Four non-overlapping lists of ten common unrelated words for recall tests

| **Word list A** | **Word list B** | **Word list C** | **Word list D** |
| --- | --- | --- | --- |
| Hotel | Sky | Woman | Water |
| River | Ocean | Rock | Church |
| Tree | Flag | Blood | Doctor |
| Skin | Dollar | Corner | Palace |
| Gold | Wife | Shoes | Fire |
| Market | Machine | Letter | Garden |
| Paper | Home | Girl | Sea |
| Child | Earth | House | Village |
| King | College | Valley | Baby |
| Book | Butter | Engine | Table |

Source: <https://www.ucl.ac.uk/drupal/site_iehc/sites/iehc/files/elsa_cognitive_booklet_w6.pdf>

Supplementary Table S2. Baseline characteristics of the sample based on the distribution of hair cortisone

|  | **Total** | **Low cortisone^a^** | **High cortisone^a^** | |
| --- | --- | --- | --- | --- |
|  |  | N (%) | N (%) | |
| ***Outcomes*** |  |  |  | |
| Worsened verbal memory at wave 6^c^ | 8.9 (±3.4) | 8.6 (±3.2) | 9.1 (±3.5) | |
| Worsened time orientation at wave 6^c^ | 0.2 (±0.5) | 0.2 (±0.4) | 0.2 (±0.5) | |
| Cumulative dementia cases |  |  |  | |
| No | 3761 (97.1) | 1938 (51.5) | 1823 (48.5) | |
| Yes | 114 (2.9) | 44 (38.6) | 70 (61.4) | |
| ***Baseline covariates at wave 6*^b^** | | | |  |
| Age^c^ | 67.8 (±9.0) | 67.4 (±8.4) | 68.2 (±9.7) | |
| Gender |  |  |  | |
| Female | 3234 (67.4) | 1809 (55.9) | 1425 (44.1) | |
| Male | 1566 (32.6) | 597 (38.1) | 969 (61.9) | |
| Ethnic |  |  |  | |
| Non-white | 90 (1.9) | 51 (56.7) | 39 (43.3) | |
| White | 4710 (98.1) | 2355 (50) | 2355 (50) | |
| Education |  |  |  | |
| No qualification | 1102 (23) | 522 (47.4) | 580 (52.6) | |
| Intermediate | 2155 (44.9) | 1116 (51.8) | 1039 (48.2) | |
| Higher education | 1543 (32.1) | 768 (49.8) | 775 (50.2) | |
| Wealth |  |  |  | |
| Quintile 1 | 737 (17.5) | 301 (40.8) | 436 (59.2) | |
| Quintile 2 | 824 (19.5) | 435 (52.8) | 389 (47.2) | |
| Quintile 3 | 878 (20.8) | 446 (50.8) | 432 (49.2) | |
| Quintile 4 | 883 (20.9) | 483 (54.7) | 400 (45.3) | |
| Quintile 5 | 896 (21.2) | 467 (52.1) | 429 (47.9) | |
| Employment |  |  |  | |
| Retired | 2907 (68.9) | 1478 (50.8) | 1429 (49.2) | |
| Employed | 1271 (30.1) | 637 (50.1) | 634 (49.9) | |
| Unemployed | 40 (0.9) | 17 (42.5) | 23 (57.5) | |
| Marital status |  |  |  | |
| Married/partner | 2760 (65.4) | 1437 (52.1) | 1323 (47.9) | |
| Separated/divorce | 513 (12.2) | 243 (47.4) | 270 (52.6) | |
| Widowed | 714 (16.9) | 335 (46.9) | 379 (53.1) | |
| Single | 231 (5.5) | 117 (50.6) | 114 (49.4) | |
| Smoking |  |  |  | |
| Never | 1688 (40.0) | 912 (54.0) | 776 (46.0) | |
| Former | 2125 (50.4) | 1060 (49.9) | 1065 (50.1) | |
| Current | 405 (9.6) | 160 (39.5) | 245 (60.5) | |
| Alcohol consumption |  |  |  | |
| Never | 535 (12.7) | 262 (49.0) | 273 (51.0) | |
| <2x/month | 1250 (29.6) | 645 (51.6) | 605 (48.4) | |
| 1-2/week | 975 (23.1) | 519 (53.2) | 456 (46.8) | |
| Daily, almost daily | 1458 (34.6) | 706 (48.4) | 752 (51.6) | |
| Physical activity |  |  |  | |
| None/light | 826 (19.6) | 356 (43.1) | 470 (56.9) | |
| Moderate | 2080 (49.3) | 1083 (52.1) | 997 (47.9) | |
| High | 1312 (31.1) | 693 (52.8) | 619 (47.2) | |
| Depression score^c^ | 1.2 (±1.7) | 1.1 (±1.6) | 1.3 (±1.8) | |
| Heart diseases |  |  |  | |
| No | 3439 (81.5) | 1782 (51.8) | 1657 (48.2) | |
| Yes | 779 (18.5) | 350 (44.9) | 429 (55.1) | |
| Stroke |  |  |  | |
| No | 4063 (96.3) | 2071 (51.0) | 1992 (49.0) | |
| Yes | 155 (3.7) | 61 (39.4) | 94 (60.6) | |
| Diabetes |  |  |  | |
| No | 3827 (90.7) | 1977 (51.7) | 1850 (48.3) | |
| Yes | 391 (9.3) | 155 (39.6) | 236 (60.4) | |
| Hypertension |  |  |  | |
| No | 2440 (57.8) | 1289 (52.8) | 1151 (47.2) | |
| Yes | 1778 (42.2) | 843 (47.4) | 935 (52.6) | |
| High cholesterol |  |  |  | |
| No | 2605 (61.8) | 1334 (51.2) | 1271 (48.8) | |
| Yes | 1613 (38.2) | 798 (49.5) | 815 (50.5) | |
| Assay phase |  |  |  | |
| Phase 1 | 2524 (52.6) | 1520 (60.2) | 1004 (39.8) | |
| Phase 2 | 2276 (47.4) | 886 (38.9) | 1390 (61.1) | |
| Hair treatment |  |  |  | |
| No | 2827 (58.9) | 1189 (42.1) | 1638 (57.9) | |
| Yes | 1973 (41.1) | 1217 (61.7) | 756 (38.3) | |

^a^For the purpose of descriptive statistics, hair cortisone was categorised based on the median into low (<6.9 pg/ml) and high (≥6.9 pg/ml).

^b^Baseline covariates at wave 6 were derived based on the sample used to investigate the association between hair cortisone and worsened verbal memory.

^c^Continuous variables were expressed as mean (±SD).


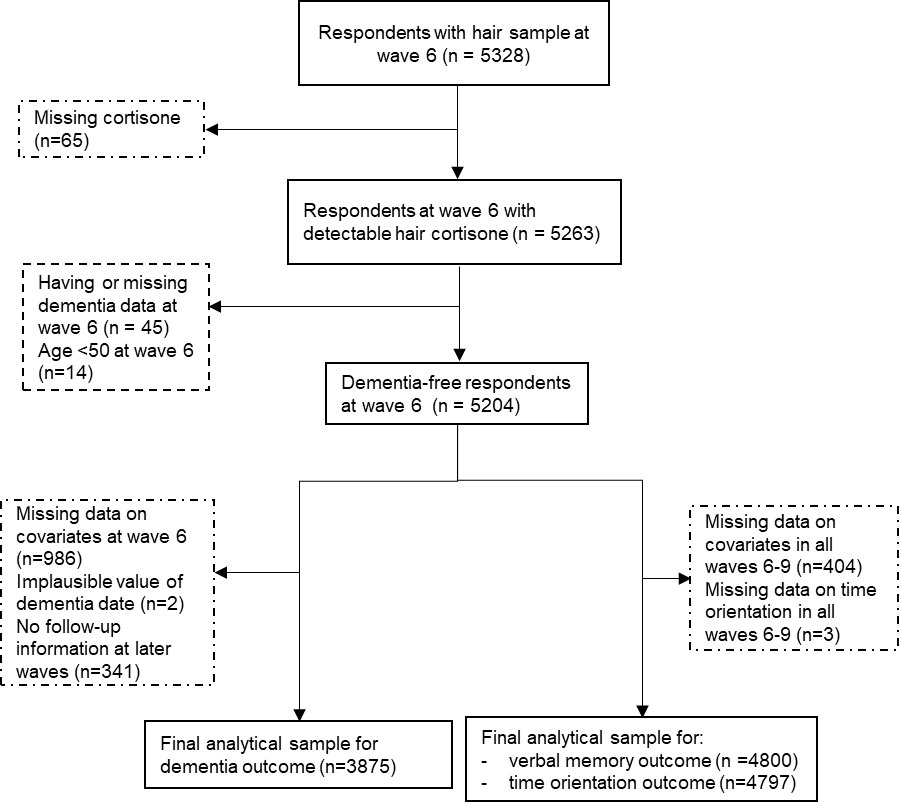


Supplementary Figure S1. Flowchart of the analytical sample of hair cortisone

Supplementary Figure S2. Associations between **(a)** hair cortisol and worsened verbal memory; **(b)** hair cortisone and worsened verbal memory; **(c)** hair cortisone and worsened time orientation after adjustment for different covariates separately


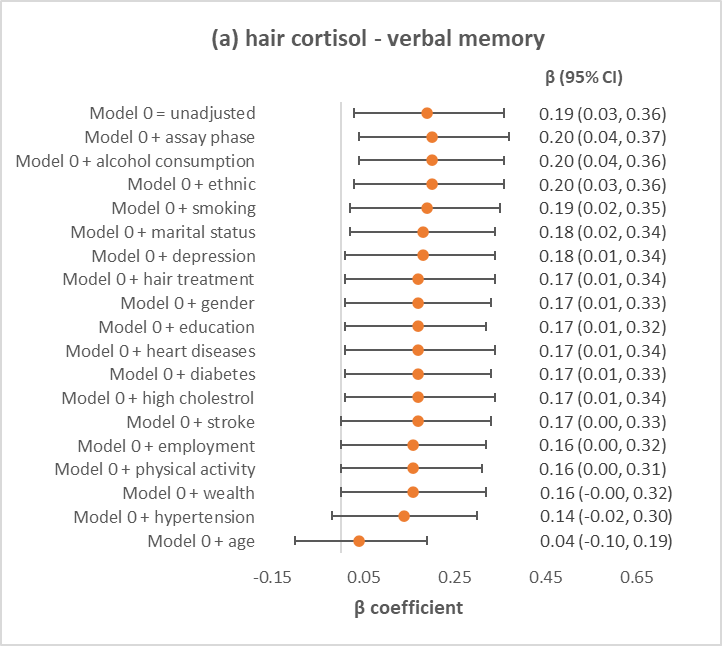

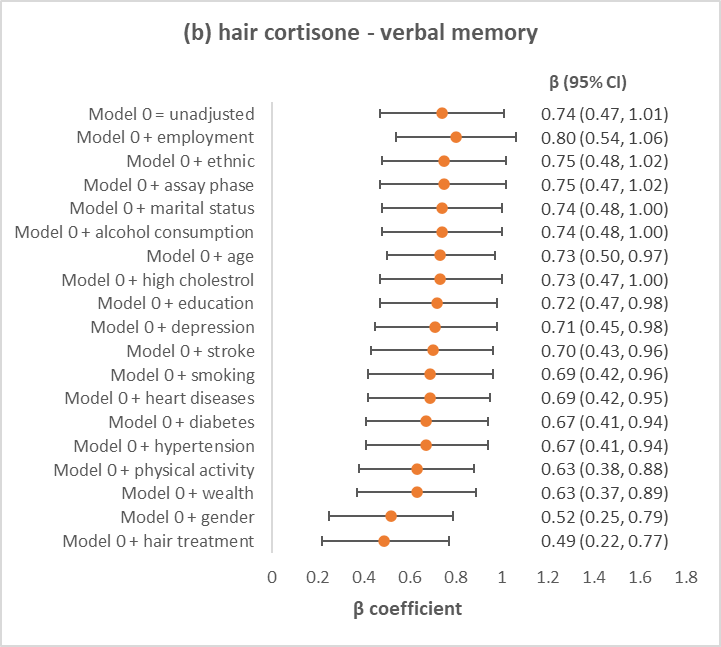

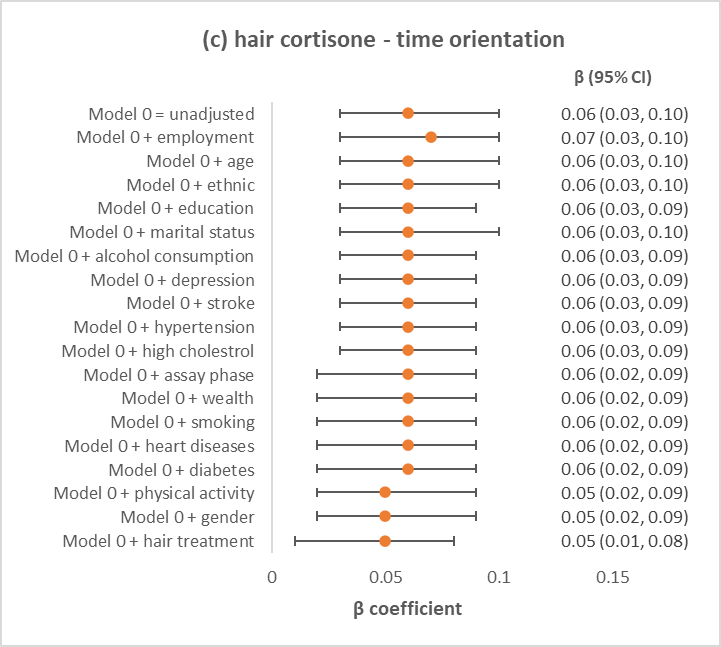

Supplement: Supplementary file 1 — Supplementary Information. [file 41598_2022_25143_MOESM1_ESM.docx]
